# A cross-sectional single-centre study on the spectrum of Pompe disease, German patients: molecular analysis of the GAA gene, manifestation and genotype-phenotype correlations

**DOI:** 10.1186/1750-1172-7-35

**Published:** 2012-06-07

**Authors:** Andreas Herzog, Ralf Hartung, Arnold J J Reuser, Pia Hermanns, Heiko Runz, Nesrin Karabul, Seyfullah Gökce, Joachim Pohlenz, Christoph Kampmann, Christina Lampe, Michael Beck, Eugen Mengel

**Affiliations:** 1 Center for Pediatric and Adolescent Medicine, University Medical Center, Langenbeckstraße 1, 55131, Mainz, Germany; 2 Department of Clinical Genetics, Erasmus MC University Medical Center, Dr Molewaterplein 50, 3015GE, Rotterdam, The Netherlands; 3 Institute of Human Genetics, University of Heidelberg, Im Neuenheimer Feld 366, 69120, Heidelberg, Germany

**Keywords:** Glycogen storage disease type II, Pompe disease, GAA, Lysosomal storage diseases, Genotype phenotype correlations, Enzyme replacement therapy

## Abstract

**Background:**

Pompe disease (Glycogen storage disease type II, GSD II, acid alpha-glucosidase deficiency, acid maltase deficiency, OMIM # 232300) is an autosomal-recessive lysosomal storage disorder due to a deficiency of acid alpha-glucosidase (GAA, acid maltase, EC 3.2.1.20, Swiss-Prot P10253). Clinical manifestations are dominated by progressive weakness of skeletal muscle throughout the clinical spectrum. In addition, the classic infantile form is characterised by hypertrophic cardiomyopathy.

**Methods:**

In a cross-sectional single-centre study we clinically assessed 3 patients with classic infantile Pompe disease and 39 patients with non-classic presentations, measured their acid alpha-glucosidase activities and analysed their GAA genes.

**Results:**

Classic infantile patients had nearly absent residual enzyme activities and a typical clinical course with hypertrophic cardiomyopathy until the beginning of therapy. The disease manifestations in non-classic patients were heterogeneous. There was a broad variability in the decline of locomotive and respiratory function. The age of onset ranged from birth to late adulthood and correlated with enzyme activities. Molecular analysis revealed as many as 33 different mutations, 14 of which are novel. All classic infantile patients had two severe mutations. The most common mutation in the non-classic group was c.-32-13 T > G. It was associated with a milder course in this subgroup.

**Conclusions:**

Disease manifestation strongly correlates with the nature of the GAA mutations, while the variable progression in non-classic Pompe disease is likely to be explained by yet unknown modifying factors. This study provides the first comprehensive dataset on the clinical course and the mutational spectrum of Pompe disease in Germany.

## Background

Pompe disease (Glycogen storage disease type II, GSD II, acid alpha-glucosidase deficiency, acid maltase deficiency, OMIM # 232300) is an autosomal-recessive lysosomal storage disorder caused by a deficiency of acid alpha-glucosidase (GAA, acid maltase, EC 3.2.1.20, Swiss-Prot P10253) [1]. Accumulation of glycogen in various tissues leads to a broad and continuous spectrum of clinical phenotypes that can be categorised according to clinical features into classic infantile and non-classic forms. Patients with non-classic disease can further be divided over those with a childhood, juvenile or adult course [1]. All patients show progressive skeletal muscle weakness which results in a decline of locomotive and respiratory functions. In addition, the classic infantile form is characterised by hypertrophic cardiomyopathy [1]. Classic infantile patients show first symptoms within the first months of life with heart failure and a profound muscle weakness. Without therapy, most of classic infantile patients die within the first year of life [2,3].

Among patients with non-classic forms complaints may begin at any time between early childhood and late adulthood [4,5]. With disease progression patients develop progressive myopathy that may result in immobilisation and respiratory insufficiency [4].

The frequency of Pompe disease varies among different ethnic groups and is estimated as 1/138,000 for classic infantile and 1/57,000 for non-classic patients in the Netherlands [1].

The GAA gene (OMIM # 606800) is located on chromosome 17q25.2-q25.3 [6]. With a length of ~20 kb it contains 20 exons and encodes for a cDNA of 3.6 kb, the start codon beginning at position 33 of exon 2 [7]. The resulting product is a protein of 952 amino acids [8].

Until now, 361 variants have been described in the GAA gene, 78 of which are polymorphisms while 248 are considered as disease-causing mutations [9]. Mutations are randomly spread over the whole gene and typically private. However, some mutations appear with considerable frequency in distinct ethnic groups. For instance, in cohorts of non-classic Caucasian patients c.-32-13 T > G is the most common mutation with a frequency of 34-47% [10-14]. This mutation leads to impaired splicing of exon 2 with about 10% of normally spliced products [15].

The clinical course of Pompe disease primarily depends on the residual acid alpha-glucosidase activity as determined by the genotype [10,16,17]. However, this correlation is afflicted with a broad variability in enzyme activities as well as in clinical signs, particularly in patients with the non-classic forms [18].

Here we describe disease presentation, enzymatic and molecular findings in a German cohort consisting of 3 patients with classic infantile Pompe disease and 39 patients with non-classic disease presentation.

## Materials and methods

### Patients and clinical data

Forty-two patients with a clinical and enzymatic diagnosis of Pompe disease were included in this study.

Patients are usually categorised with respect to age of onset, extent of organ involvement and disease progression [1]. We distinguished patients with a classic infantile course that are severely affected by hypertrophic cardiomyopathy, onset in the first month of life and rapid progression from those with a non-classic course with predominant muscle involvement but without hypertrophic cardiomyopathy. Non-classic disease includes the childhood, juvenile and adult forms, which are not unambiguously distinguished in the literature. We therefore defined the childhood forms by having motor delay and progression of muscle weakness before achievement of motor milestones. The juvenile forms were defined by progression of muscle weakness after achievement of motor milestones but before the end of growth. Adult forms were defined by having a progression of muscle weakness not until the end of growth. It is of note that non-classic patients may have an earlier onset followed by temporary regression of symptoms before muscle weakness becomes progressive.

In non-classic patients all data were collected in the observation period before starting enzyme replacement therapy, when they only received supportive care. Since we initiated enzyme replacement therapy with alglucosidase alpha in all classic infantile patients shortly after referring to our centre, data on their natural course are limited and amended by a description of the course under ERT.

Clinical data were retrospectively collected from health records. Assessment included physical examination, manual muscle testing using the Medical Research Council (MRC) grading scale for different muscle groups, the Walton & Gardner-Medwin scale, and the per cent predicted forced vital capacity (FVC) in sitting and supine position. A total MRC-score was defined as the mean of MRC-grades of neck flexion, shoulder abduction, elbow flexion, hip flexion, knee extension and foot extension. Echocardiography was performed as previously described [19].

Patient 42 suffers from a 2q37 deletion syndrome in addition to Pompe disease (unpublished). Since it cannot be excluded that muscular hypotonia in this patient is caused by genetic factors other than pathogenic variants in the GAA gene this patient was excluded from clinical characterisation [20].

Since parents of patients 2 and 3 are consanguineous, there are four independent alleles in classic infantile and 73 independent alleles in non-classic patients.

All patients were Caucasians, patients 2 and 3 having a Turkish, the others having a German ancestry.

### Biochemical assays

In 32 of the 42 patients GAA activity was measured in isolated lymphocytes of fresh blood samples with 4-methylumbelliferyl-α-D-glucopyranoside as substrate and acarbose as an inhibitor of interfering maltase-glucoamylase activity according to a standardised protocol [21,22]. The GAA activity was expressed in nmol hydrolysed 4-MU-αGlc/hour/mg protein (nmole/mg/hr). In 7 patients GAA activity was measured according to a former protocol in isolated lymphocytes with 4-methylumbelliferyl-α-D-glucopyranoside as substrate without acarbose and in 3 patients enzyme activities were measured in external laboratories. Those enzyme activities were not included in further analysis.

### Mutation analysis

Direct sequencing of the GAA gene was performed using standard procedures (Additional file 1). Exons of the gDNA were amplified using long range PCRs of eight fragments of the GAA gene. PCR of cDNA was performed in overlapping fragments as described previously [23]. To be able to detect or exclude large deletions that escape routine Sanger sequencing long range PCRs over more fragments were carried out.

Obtained sequences were compared with the reference sequences NM_000152.3 and NC_000017.9. The A of the start codon, defining position +1 of cDNA, lies at positions 3032 and 368 of those sequences, respectively. The ATG codon represents +1 of the amino acid numbering according to NP_00143.2. Variants were described according to the guidelines of the Human Genome Variation Society [24]. All nucleotide differences between the patients and the reference sequence were compared to the GAA mutation database of the Erasmus MC University Medical Center Rotterdam and to the dbSNP database of the National Center of Biological Information [9,25]. Variants that were listed as polymorphisms or assured disease causing mutations in the GAA mutation database were not further characterised. Novel nonsense mutations as well as frameshift mutations leading to a premature stop codon were assumed to be disease causing if the stop codon was lying upstream of another known disease causing nonsense mutation. All other mutations were classified as potentially disease causing and were further analysed.

The pathogenic nature of novel missense mutations was verified by restriction digestion or direct sequencing of 120 alleles of unaffected individuals. Furthermore an alignment with orthologous sequences was performed.

To identify transcriptional products of splice site mutations, PCR products were cloned into an expression vector system. We purified cDNAs using the peqGOLD MicroSpin Gel Extraction Kit (Peqlab, Erlangen, Germany) and cloned them into the pGEM®-T vector (Promega, Mannheim, Germany). After transformation into XL10-Gold® Ultracompetent Cells (Stratagene, Waldbronn, Germany) positive clones were selected and plasmid DNA isolated using the Zyppy™ Plasmid Miniprep Kit (Zymo research, Freiburg, Germany).

### Statistical analysis

Descriptive statistical analysis was performed using SPSS Statistics version 17.0 (SPSS Software, München, Germany). Since clinical data were not comprehensive in all patients, analysis was done with list-wise deletion of missing values.

### Ethics

This study was approved in the context of the Pompe Registry by the ethics committee of Landesärztekammer Mainz, Germany.

## Results

### Disease manifestations in classic infantile patients

Patient 1 was born after an inconspicuous pregnancy with Cesarean section in the 38^th^ week of gestation because of a silent cardiotocogram with a birth weight of 3.6 kg (Table 1, Table 2). Postpartum she had a respiratory distress syndrome that required brief ventilation with facemask. On day three after birth she had myocloni and a periodic breathing with reduced blood oxygen saturation. In further course she developed a progressive muscular hypotonia and dystonia with reduction of movements. Left ventricular hypertrophy was diagnosed at the age of 2.5 month. Diagnosis was made at the age of 3 month. At the start of enzyme replacement therapy at the age of 5 month, she had generalised muscular hypotonia as well as hypertrophic cardiomyopathy.

**Table 1:** Clinical, enzymatic and molecular information on 42 German patients with Pompe disease

**Patient**	**Age at onset [years]**	**First symptoms**	**Walton & Gardner-Medwin scale**	**FVC in sitting/ supine position [%]**	**GAA activity [nmole/mg/hr]**	**Allele 1**	**Allele 2**	**Observation period [years]**	**Gender**
**classic infantile**									
1	postnatal	respiratory insufficiency	-	-	1.0^$^	c.1799G>A	c.2481+102_2646+31del	6	f
2	prenatal	cardiomegaly	-	-	0.3^$^	c.1637-2A>G	c.1637-2A>G	2	m
3	3/12	adynamia in breathing and sucking	-	-	1.2^$^	c.[2740dup; 2742dup]	c.[2740dup; 2742dup]	4/12	f
**childhood**									
4	1	no standing aged 1 year	3	48/50	0.01^§^	c.-32-13T > G	c.1051delG	9	m
5	1.5	no free walking aged 1.5 years	8 (walking support aged 15 years, wheelchair aged 17 years)	?	0.2^$^	c.2297A>G	c.1561G>A	29	f
6	1.5	no free walking aged 1.5 years	3	32/27	2.0^$^	c.1370C>T	c.1128_1129delinsC	4	m
**juvenile**									
7	1/12	adynamia in breathing and swallowing	0	75/78	2.1^$^	c.-32-13T > G	c.2481+102_2646+31del	14	m
8	0	muscular hypotonia, adynamia in swallowing	7 (wheelchair aged 19 years)	32	^#^	c.-32-13T > G	c.525delT	21	f
9	1	no standing aged 1 year	2	72/54	0.8^$^	c.-32-13T > G	c.2481+102_2646+31del	30	m
10^γ^	1	no crawling aged 1 year	0	71/66	0.5^$^	c.-32-13T > G	c.525delT	15	m
11	1	muscle weakness, adynamia in swallowing	3	80/74	1.2^$^	c.-32-13T > G	c.1548G>A	20	f
12	2	muscle weakness	0	89/86	0.5^$^	c.-32-13T > G	c.118C>T	20	m
13	2	muscle weakness	2	?	0.02^§^	c.-32-13T > G	c.2214G>A	2	f
14	6.5	muscle weakness	0	91/94	0.03^§^	c.-32-13T > G	c.1561G>A	5	m
15	3	developmental retardation, abnormal gait	2	28/20	0.02^§^	c.-32-13T > G	c.2738C>G	20	m
16	13	muscle weakness	0	82/75	0.9^$^	c.-32-13T > G	c.-32-13T > G	13	m
17	9	limb girdle weakness	7 (wheelchair aged 33 years)	56/42	2.8^$^	c.1076-22T > G	[c.1426C>A; c.1437+1G>A]	34	f
18^β^	12	limb girdle weakness	0	98/90	0.3^$^	c.-32-13T > G	c.307T > G	12	f
19	12	limb girdle weakness	0	108/105	1.0^$^	c.-32-13T > G	c.1143delC	25	m
20	13	muscle weakness	3	50/46	0.3^$^	c.2014C>T	c.1703A>T	25	f
21	15	muscle weakness	0	65/60	1.1^$^	c.-32-13T > G	c.1441T>C	17	m
22^β^	16	muscle weakness	3	69/58	1.2^$^	c.-32-13T > G	c.307T > G	19	f
**adult**									
23	12	weakness of trunk muscles, scoliosis	4	61/36	0.04^§^	c.-32-13T > G	c.1799G>A	48	m
24	18	muscle weakness	3	50/25	1.0^$^	c.-32-13T > G	c.1143delC	46	m
25	18	muscle weakness	2	53/30	1.2^$^	c.-32-13T > G	c.701C>A	52	m
26	20	limb girdle weakness	3	71/46	3.9^$^	c.-32-13T > G	c.877G>A	44	f
27^α^	23	limb girdle weakness	4 (walking support aged 33 years)	76/68	0.03^§^	c.-32-13T > G	c.1291_1299delCTGCACCAG	35	m
28	27	limb girdle weakness	6 (walking support aged 51 years)	33/26	4.7^$^	c.-32-13T > G	c.1802C>T	53	m
29^α^	29	limb girdle weakness	0	101/81	0.09^§^	c.-32-13T > G	c.1291_1299delCTGCACCAG	42	f
30	30	limb girdle weakness	3	98/90	1.2^$^	c.-32-13T > G	c.525delT	47	f
31	30	limb girdle weakness	3	66/25	1.8^$^	c.-32-13T > G	c.2608C>T	46	f
32	30	backache	1	?	0.8^$^	c.-32-13T > G	c.1005_1006insGG	70	m
33	35	limb girdle weakness	3	43/19	4.1^$^	c.-32-13T > G	c.1564C>G	45	m
34	35	limb and shoulder girdle weakness	1	124/105	4.9^$^	c.-32-13T > G	c.307T > G	37	f
35	38	limb girdle weakness	6 (walking support aged 57 years)	33/18	^#^	c.-32-13T > G	c.2214G>A	63	f
36	40	limb girdle weakness	6 (walking support aged 59 years)	50/37	1.1^$^	c.-32-13T > G	c.2205_2206insT	70	f
37	47	limb girdle myalgia	3	120/119	2.8^$^	c.-32-13T > G	c.2322_2323insGGTGAGTCTGCAAACGGGGAGT	59	f
38	48	limb girdle weakness	6 (walking support aged 62 years)	87/61	0.7^$^	c.-32-13T > G	c.877G>A	62	f
39	56	limb girdle weakness, backache	2	120/114	1.7^$^	c.-32-13T > G	c.2237G>A	69	f
40	?	?	?	?	1.5^$^	c.-32-13T > G	c.1687_1688insCACC	65	f
**asymptomatic**									
41^γ^	-	asymptomatic	0	95/98	0.4^$^	c.-32-13T > G	c.525delT	20	f
**excluded from clinical analysis**									
42	-	-	-	-	^#^	c.-32-13T > G	c.2481+102_2646+31del	1	m

^α, β^ siblings

^γ^ first degree cousins.

^$^ GAA activity measured in isolated lymphocytes with 4-methylumbelliferyl-α-D-glucopyranoside as substrate and acarbose (normal range of 9-42 nmole/mg/hr).

^§^ GAA activity measured according to a former protocol in isolated lymphocytes with 4-methylumbelliferyl-α-D-glucopyranoside as substrate without acarbose (normal range of 0,19-0,45 nmole/mg/hr). Those enzyme activities were not included in further analysis.

^#^ no own data available.

**Table 2:** Disease course in 3 classic infantile patients

**Patient 1**	
**Age**	**Disease Course**
≤ 0	inconspicuous pregnancy, Cesarean section in the 38^th^ week of gestation because of a silent cardiotocogram, birth weight of 3.6 kg, respiratory distress syndrome that required brief ventilation with face mask postpartum
3 days	myocloni and a periodic breathing with decreased blood oxygen saturation
2.5 month	hypotonia, dystonia, reduction of movements, persistent increase of ASAT, ALAT and CK, diagnosis of hypertrophic cardiomyopathy
5 month	generalised muscular hypotonia, movement of extremities possible, hypertrophic cardiomyopathy start of enzyme replacement therapy
11 month	first free sitting with arm support
16 month	first rolling, tendency to pull to stand, active crawling, IVSd 7.7 mm [2.6 - 5.6]^§^
22 month	beginning of four point kneeling and crawling, begins supported standing, IVSd 6.9 mm [2.7 – 5.9]
2 4/12 years	sitting without arm support with proper head control, supported standing with reduced force of Mm. quadriceps femoris, IVSd 6.6 mm [2.7 – 5.9]
4 years	Unclear speech, stands on hand and feet, and sits unsupported, crawling, good arm control but poor head control, and hypotonic upper body, IVSd 6.7 mm [3.3 – 6.3]
5 years	start of invasive ventilation in supine position because of atelectasis, normalized cardiac function with IVSd 6 mm [3.3 – 6.3]
**Patient 2**	
**Age**	**Disease Course**
≤ 0	normal prenatal development despite of developing a hypertrophic cardiomyopathy, spontaneous delivery in the 37th week of gestation, birth weight of 3.1 kg
4 days	start of enzyme replacement therapy, IVSd 6.7 mm [2.3 – 4.9]
2 month	head control for 2-3 sec. when pulled up from supine position, IVSd 6.6 mm [2,4 – 5,2]
5 month	normal development, normal force, lifts head actively in prone position, rolling from prone to supine position and back, IVSd 5 mm [2.5 – 5.3]
8 month	normal development, supported standing, crawling, free sitting, normal force, IVSd 6 mm [2.6 – 5.6]
11 month	independent walking and standing
15 month	normal development, normal force, walking but a bit waddling, discrete Facies hypotonica, proper standing up without Gower´s sign, IVSd 5 mm [2.6 – 5.8]
**Patient 3**	
≤ 0	inconspicuous pregnancy, spontaneous delivery in the 39th week of gestation, birth weight of 3.2 kg
2 month	weak in breathing and sucking
3 month	generalised muscular hypotonia with respiratory insufficiency, cyanosis, hypertrophic cardiomyopathy, IVSd 10 mm [1.4 – 3.8] start of enzyme replacement therapy
data on the further course are not yet available	

Patient 3 was born spontaneously in the 39^th^ week of gestation with a birth weight of 3.2 kg and developed initially normal. Aged 2 month she was weak in breathing and sucking. She developed generalised muscular hypotonia with respiratory insufficiency as well as hypertrophic cardiomyopathy.

The diagnostic delay in these two patients was 3 and 1 month, respectively.

The diagnosis in patient 2 was prenatally established and was based on the finding of hypertrophic cardiomyopathy. The prenatal development was otherwise normal and the baby boy was born spontaneously in the 37^th^ week of gestation with a birth weight of 3.1 kg. Enzyme replacement therapy was started at the fourth day of life when the baby boy was still asymptomatic. The intervention resulted in a regression of cardiomyopathy and enabled a nearly asymptomatic life until the end of the 2 years follow-up period.

### Disease manifestations in non-classic patients

**Figure 1:**
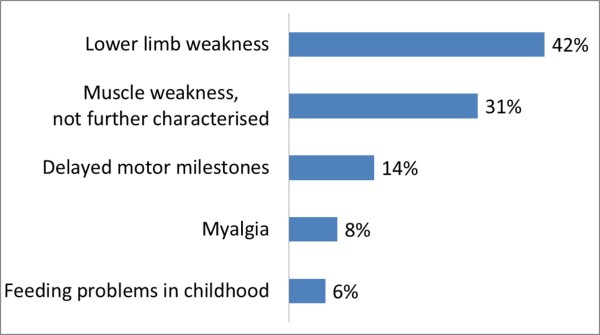
**Frequency of first symptoms in non-classic patients.** About one third of the patients had mild symptoms in childhood preferentially consisting of delayed motor milestones and feeding problems.

The observation period of the included 37 symptomatic non-classic patients ranges from 1.5 to 70 years with a median of 30 (Quartiles: 18, 47) years. Age at onset is known in 36 of them and spans from birth to the age of 56 years with a median of 14 (Quartiles: 2, 30) years. The first symptoms of these patients are categorised in Figure 1.

In four patients it came to a temporary regression of symptoms in childhood or adolescence before their muscle weakness became progressive. Patient 8 had had an early disease onset with postnatal muscular hypotonia and adynamia in swallowing. Her muscular weakness had improved until she developed a proximal muscle weakness at the age of 8 years, which was rapidly progressive and led to wheelchair dependence at the age of 19 years. Patient 9 also had an early onset disease with delayed motor milestones. He reached standing at one year of age and was able to walk aged 3 years. He did well until the age of 17 years, when he was resilient and active, before muscle weakness progressed. Patient 12 suffered from generalised muscular hypotonia at the age of 2 years. His fitness considerably improved during childhood and has remained stable over the observation period until the age of 20 years. Patient 23 manifested weakness of trunk muscles and scoliosis at the age of 12 years, but his fitness improved till the age of 25 years while he was active in sports. Progressive muscle weakness began in his middle twenties and proceeded until the end of the observation period.

Patients were diagnosed between 5 month and 66 years of age with the median age of 22 (Quartiles: 4, 44) years (n = 36). The median diagnostic delay in symptomatic patients was 8 (Quartiles: 2, 15) years, ranging from 0 to 34 years (n = 36).

**Figure 2:**
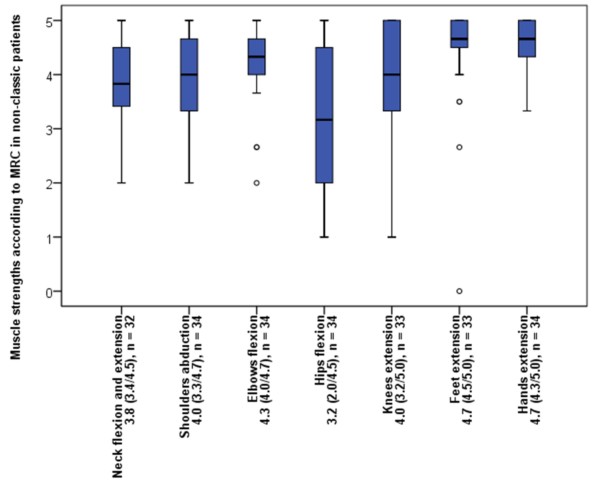
**Muscle strength of different muscle groups in non-classic patients according to MRC. ** The median muscle strength shows a predominant involvement of proximal muscles. The lower limbs are most affected.

At the end of the observation period 29 of 37 included symptomatic patients (78%) were able to walk without any assistance while 5 (14%) needed a supportive device and 3 (8%) a wheelchair. Walking assistance was first used at the median age of 54 (Quartiles: 28, 60) years ranging from 15 to 62 years (n = 6). Disease duration until usage of walking assistance had a median of 19 (Quartiles: 12, 21) years ranging from 10 to 24 years (n = 5). The 3 patients using a wheelchair became dependent on it at the age of 17, 19 and 33 years after disease duration of 16, 19 and 24 years. The mobility of each patient graded according to the Walton & Gardner-Medwin scale is shown in Table 1. The median muscle strength of different muscle groups according to MRC is shown in Figure 2.

Patient 5 needed continuous invasive ventilation since the age of 27 years after disease duration of 26 years. Eight patients (22%) used non-invasive ventilation while lying. Non-invasive ventilation was started at the median age of 46 (Quartiles: 21, 48) years ranging from 19 to 63 years (n = 9) after disease duration of 21 (Quartiles: 17, 27) years ranging from 11 to 29 years (n = 9). FVC in sitting position had a median value of 71% (Quartiles: 50, 92) of normal with a range of 28 to 124% (n = 34). It decreased to 60% (Quartiles: 33, 88) in the supine position with a range of 18 to 119% (n = 33).

To further explore disease progression we analysed the correlations between clinical parameters and age as well as disease duration that are given in Table 3.

**Table 3:** Correlation of clinical parameters with age and disease duration in patients with non-classic disease

	**Correlation with age**		**Correlation with disease duration**	
	**Spearman-Rho**	**Significance**	**Spearman-Rho**	**Significance**
**Total MRC-score**	-0.491	0.006	-0.543	0.002
**Walton & Gardner-Medwin Scale**	0.451	0.005	0.481	0.003
**FVC supine**	-0.214	0.231	-0.507	0.003
**FVC sitting**	0.001	0.994	-0.399	0.021

### Acid alpha-glucosidase activity and clinical course

**Figure 3:**
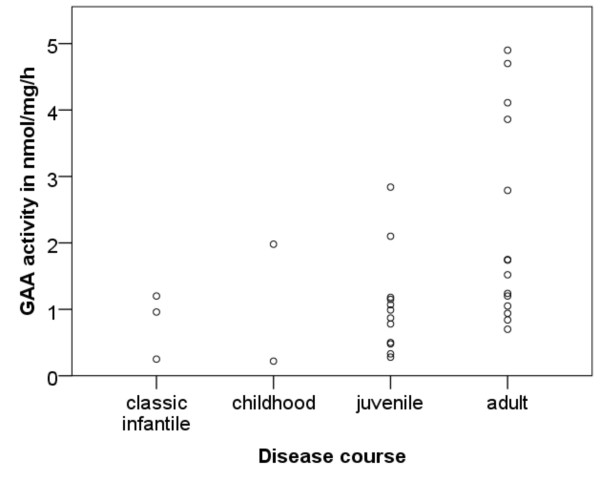
Correlation between disease course and residual acid alpha-glucosidase activity in lymphocytes.

Enzyme activities in lymphocytes of the three classic infantile patients were 1.0, 0.3 and 1.2 nmole/mg/hr (Normal range 9–42 nmole/mg/hr). Residual activities of 30 non-classic patients ranged from 0.2 to 4.9 nmole/mg/hr with a median of 1.1 (Quartiles: 0.8, 2.0) nmole/mg/hr. Figure 3 shows the enzyme activities in the different courses of non-classic disease. The Wilcoxon-Mann–Whitney-test revealed significant higher activities in patients with an adult course than in patients with a juvenile course (p = 0.016).

In the group of non-classic patients there was a trend towards later onset of symptoms with higher enzyme activities (r_s_ = 0.354, p = 0.070). No correlation was found between the enzyme activity and the current disease severity (i.e. the sum of muscle strength according to the MRC for the different muscle groups and the forced vital capacity).

### Serum enzymes

Serum values of creatine kinase (CK), lactate dehydrogenase (LDH), alanine aminotransferase (ALAT) and aspartate aminotransferase (ASAT) were elevated in nearly all patients. Their distribution is shown in Table 4.

**Table 4:** Serum enzymes of 2 patients with classic infantile and 29 patients with non-classic disease

	**CK [U/l]**	**LDH [U/l]**	**ALAT [U/l]**	**ASAT [U/l]**
**Reference values**	30-200	< 245	< 50	5-35
**Median**	607	363	94	82
**Minimum**	148	242	40	25
**25th percentile**	388	302	62	64
**75th percentile**	899	476	188	154
**Maximum**	2256	1416	548	532

There were not found significant differences in serum enzymes depending on the different courses of disease.

### Mutation spectrum

**Figure 4:**
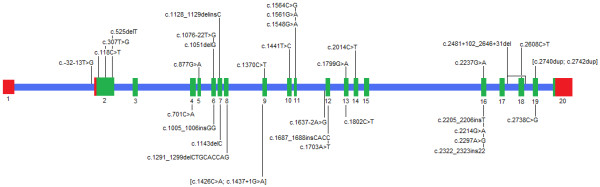
Mutation spectrum in 42 German patients. Previously described mutations are shown above and new mutations below the diagrammed GAA gene (red: URT, blue: introns, green: exons).

Mutations were identified in each of the 77 alleles (Table 1). Figure 4 shows their random distribution over the GAA gene, Table 5 their frequency and predicted effect [9,25,26]. Of the 33 different variants that we identified, 14 (42%) had not been reported previously.

**Table 5:** Frequency and predicted effect of identified mutations

**Mutation**	**Effect on cDNA or protein**	**Predicted severity**[9,25,26]	**Frequency**
c.-32-13T>G	impaired splicing of exon 2	mild	34/73 alleles in non-classic patients
c.118C>T	p.Arg40X	severe	1/42 alleles at risk
c.307T>G	p.Cys103Gly	severe	2/42 alleles at risk
c.525delT	p.Glu176fsX45	severe	3/42 alleles at risk
***c.701C>A***	***p.Thr234Lys***	***potentially less severe***^α^	***1/42 alleles at risk***
c.877G>A	p.Gly293Arg	severe	2/42 alleles at risk
***c.1005_1006insGG***	***p.Ile336GlyfsX56***	***severe***	***1/42 alleles at risk***
c.1051delG	p.Val351CysfsX41	severe	1/42 alleles at risk
c.1076-22T>G	p.[Asp319_Val358delinsGlySerArgArgTrpProAla; Gly334_Val358delinsGlySerArgArgTrpProAla]	mild	1/73 alleles in non-classic patients
c.1128_1129delinsC	p.Trp376CysfsX16	severe	1/42 alleles at risk
***c.1143delC***	***p.Thr381fsX10***	***severe***	***2/42 alleles at risk***
***c.1291_1299delCTGCACCAG***	***p.Leu431_Gln433del***	***unknown***	***1/77 alleles***
c.1370C>T	p.Pro457Leu	mild	1/73 alleles in non-classic patients
***c.[1426C>A; 1437+1G>A]***	***r.1327_1437del p.Asp443_Lys479del***	***unknown***	***1/77 alleles***
c.1441T>C	p.Trp481Arg	severe	1/42 alleles at risk
c.1548G>A	p.Trp516X	severe	1/42 alleles at risk
c.1561G>A	p.Glu521Lys	severe	2/42 alleles at risk
c.1564C>G	p.Pro522Ala	severe	1/42 alleles at risk
***c.1637-2A>G***	***r.[1637_1659del; 1637_1682del; 1637_1738del; 1637_1754del; [1637_1659del, 1755-110_1755-1ins]] p.[V547GfsX80; G546AfsX16; G546_E579del; V547RfsX2; V547_R585delinsGGHHLCLQPPVSLHTLQPAQPLRPDRSHRLPOPLPHPRKLLAPSSALLVTGFPSPPAPHSPHGVPHHPR]***	***severe***	***1/42 alleles at risk***
***c.1687_1688insCACC***	***p.Gln563ProfsX73***	***severe***	***1/42 alleles at risk***
***c.1703A>T***	***p.His568Leu***	***unknown***	***1/77 alleles***
c.1799G>A	p.Arg600His	severe	2/42 alleles at risk
***c.1802C>T***	***p.Ser601Leu***	***potentially less severe***^α^	***1/77 alleles***
c.2014C>T	p.Arg672Trp	intermediate	1/77 alleles
***c.2205_2206insT***	***p.Ser736Xfs***	***severe***	***1/42 alleles at risk***
***c.2214G>A***	***p.Trp738X***	***severe***	***2//42 alleles at risk***
c.2237G>A	p.Trp746X	severe	1/42 alleles at risk
***c.2297A>G***	***p.Tyr765Cys***	***unknown***	***1/77 alleles***
***c.2322_2323insGGTGAGTCTGCAAACGGGGAGT***	***p.Leu775GlyfsX70***	***severe***	***1//42 alleles at risk***
c.2481+102_2646+31del	p.Gly828_Asn882del	severe	4//42 alleles at risk
c.2608C>T	p.Arg870X	severe	1//42 alleles at risk
***c.2738C>G***	***p.Pro912Arg***	***unknown***	***1/77 alleles***
c.[2740dup; 2742dup]	p.Gln914fsX30	severe	1//42 alleles at risk

^α^ unpublished data, that will be included in the upcoming update of the Pompe disease mutation database

novel mutations in bold italics.

### Novel mutations

In total, we identified 14 novel mutations. Six of them lead to a premature stop in protein synthesis (c.1005_1006insGG, c.1143delC, c.1687_1688insCACC, c.2205_2206insT, c.2214 G > A and c.2322_2323insGGTGAGTCTGCAAACGGGGAGT). One splice site mutation c.1437 + 1 G > A occurring ‘in cis’ with c.1426 C > A (p.Leu476Met) was found compound heterozygous with the intronic mutation c.1076-22 T > G, also causing aberrant splicing. Cloning of the transcriptional products into an expression vector system demonstrated that there were no normally spliced products derived from the c. [1426 C > A; 1437 + 1 G > A] allele. The alternatively spliced products derived from the c.1076-22 T > G allele had the wild type cytosine at position c.1426. Thus the variants at position c.1426 C > A (p.Leu476Met) and c.1437 + 1 G > A are located on the same allele. In the solely identified splice product of the c. [1426 C > A; 1437 + 1 G > A] allele exon 9 was skipped (r.1327_1437del), which results in a protein bearing the in frame deletion p.Asp443_Lys479del.

The third splice site mutation c.1637-2A > G also appeared to fully prevent correct splicing. Instead, 5 alternative splice products could be cloned all of which lack the 5´-end of exon 12. Amplification of cDNA with CTG GAG GGT CCC CCC AAC CAC C as reverse primer located in this region did not reveal any products.

Furthermore, five novel missense mutations and one deletion of 9 nucleotides were identified (c.701 C > A (pThr234Lys) c.1703A > T (p.His568Leu), c.1802 C > T (p.Ser601Leu), c.2297A > G (p.Tyr765Cys), c.2738 C > G (p.Pro912Arg) and c.1291_1299delCTGCACCAG (p.Leu431_Gln433del)). These mutations were not encountered among 120 alleles of unaffected persons. The corresponding amino acids are highly conserved among all compared eutheria (Additional file 2).

### Genetic variability

We analysed 34 SNPs located within the sequenced areas and compared their frequencies to those of reference populations (Additional file 3) [9,25,27].

In patients bearing the c.-32-13 T > G mutation the variants c.324 C > T (p.Cys108Cys), c.596 G > A (p.Arg199His), c.668A > G (p.His223Arg), c.1203A > G (p.Gln401Gln), c.1327-18 G > A, c.2040 + 20 G > A and c.2338A > G (p.Ile780Val) appear considerably more frequent than in reference collectives (Hapmap-CEU, AoD Caucasian). In patients not bearing c.-32-13 T > G the frequencies of these polymorphisms are similar to those of the reference collectives.

In the 5´-untranslated region of the GAA gene we identified the novel polymorphism c.-367-157 C > G on 9/52 alleles of the patients bearing the c.-32-13 T > G mutation.

### Genotype-phenotype correlations

**Figure 5:**
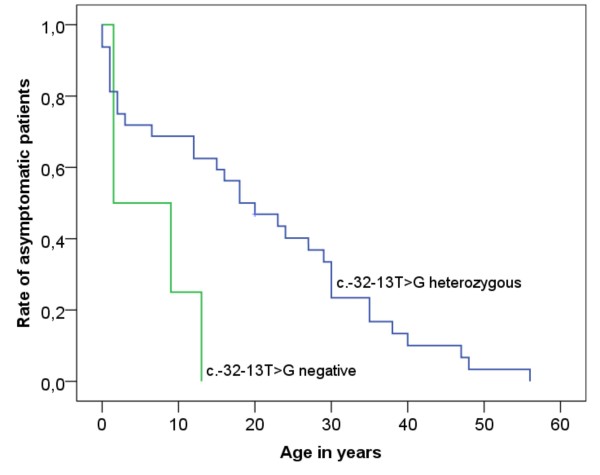
Kaplan-Meier function of age at disease onset depending on the prevalence of the common mutation c.-32-13 T > G.

All three patients with classic infantile Pompe disease had two severe mutations. In the patients with non-classic disease we analysed the disease manifestation according to the prevalence of the common c.-32-13 T > G mutation. Patient 16 is homozygous for c.-32-13 T > G as was confirmed by demonstrating heterozygosity of both his parents. He attracted attention at the age of 13 years by a reduced resilience with a slight muscle weakness. In c.-32-13 T > G genetic compounds the Log-Rank test showed a later onset compared to patients with other mutations (p = 0,025) (Figure 5). The age at onset of c.-32-13 T > G genetic compounds was 18 (Quartiles: 2, 30) years ranging from 0 to 56 years (n = 31). Four patients bearing two other mutations had first symptoms at the age of 1.5, 1.5, 9 and 13 years respectively. In summary the c.-32-13 T > G mutation is associated with a later disease manifestation.

## Discussion

### Disease manifestations in classic infantile patients

Until the start of ERT, patient 1 (at 5 month of age) and patient 3 (at 3 month of age) had a characteristic course with hypertrophic cardiomyopathy and respiratory problems resulting from muscular hypotonia developing in the first month of life [3,28]. The hypertrophic cardiomyopathy of patient 2 was discovered prenatally and ERT was started on day 4 after birth. This early intervention resulted in normalisation of the left ventricular wall thickness and a near normal development of the child up to the age of 2 years, in contrasts to the natural course of disease whereby higher motor milestones are not reached. Notably, similar effects have been reached by newborn screening and immediate initiation of ERT in 5 patients with classic infantile Pompe disease from Taiwan [29]. ERT in patient 1, who was already symptomatic at the start of therapy, resulted in a reduction of the left ventricular wall thickness to normal values and delayed, but not prevented, the occurrence of typical symptoms. This confirms previous findings that the outcome of enzyme replacement therapy depends on the clinical status of the patient at start of treatment and underlines the relevance of an early diagnosis followed by an immediate intervention [30].

### Disease manifestations in non-classic patients

There is a broad heterogeneity in the clinical course of non-classic Pompe disease with onset between birth and late adulthood. In our study the median age of onset was considerably younger than in previous studies [4,13]. This is explained in part by the fact that we have interpreted any symptom that probably relates to Pompe disease as disease onset while in other studies the beginning of progressive muscle wasting was mostly taken as first symptom. Accordingly the spectrum of first symptoms differs. Though muscle weakness is the most frequent initial symptom, other complaints like delayed achievement of motor milestones or feeding problems in childhood appear rather common [4,13]. With regard to the diagnostic delay of 8 years these findings emphasise the necessity of being aware that non-classic forms of Pompe disease can manifest in the first years of life. Additional sensitive hints can be given by serum values of CK, LDH, ALAT and ASAT that were clearly elevated in nearly all patients. Elevations of LDH, ASAT and ALAT in daily diagnostic routine can point to a muscle disease and should be further investigated by determining the CK even in asymptomatic patients. Since the efficacy of enzyme replacement therapy depends on the clinical state at start of therapy, it is important to diagnose patients before the disease has progressed to muscle wasting [31]. Remarkably, none of our patients initially had respiratory complaints in contrast to other studies wherein approximately one third of the patients had respiratory symptoms before locomotive problems manifested [5,13]. This might be explained by a difference in data acquisition, whereby we extracted the first symptoms from the patients’ health reports. The absence of subjectively sensed disorders does not exclude the prevalence of a relevant respiratory insufficiency.

Progressive muscle wasting was the salient clinical feature. The characteristic pattern of involvement of different muscle groups described previously could be confirmed [13,32]. There is a clear increase of involvement from the distal to the proximal muscles while the lower limbs are more affected than the upper limbs. Reduced strength of skeletal muscles leads to impaired walking and breathing, but the range of functional loss is very broad. Reduction of force and muscle function were correlated with age and disease duration and forced vital capacities were correlated with disease duration showing the progressive course of Pompe disease.

### Mutation analysis

Since molecular defects causing Pompe disease are numerous with most mutations appearing only sporadically, sequencing is the most appropriate method for their analysis [16,33]. To prevent the missing of large deletions we analysed both genomic DNA as well as cDNA in large fragments. By this we reached a sensitivity of 100%. Furthermore, we investigated all newly discovered sequence variants for their pathogenic nature in order to exclude false positive identification.

### Mutation spectrum

Our study underlines the enormous heterogeneity at the GAA locus that has come to light in a number of studies that were performed over the past ten years. As much as 73% (24 out of 33) of all mutations that we identified in our present study were only encountered once [9-14,33-35].

The very frequent observation of the c.-32-13 T > G mutation among the German patients studied (34 of the 38 children and adults) corresponds with the previously reported high frequency of this mutation in patients from Central and Southern Europe [10-14]. We also encountered the deletions of exon 18 (c.2481 + 102_2646 + 31del) and c.525delT in several patients, but these two mutations seem not as common in the German as in the Dutch population [10-14,34,35]. Furthermore, our studies confirmed that the missense mutations c.307 T > G and c.877 G > A are probably more frequent in Germany than in other European countries [14,16,35].

### Novel mutations

All the protein truncating mutations that were identified in this study are assumed to be deleterious since their stop codons are located upstream of those that are known to result in a complete loss of enzyme activity [9].

The complex mutation c.[1426 C > A; 1437 + 1 G > A] leads to exon 9 skipping. The concurrence of this mutation together with the mild splice site mutation c.1076-22 T > G in a patient with onset of symptoms at the age of 9 years and wheelchair dependency at the age of 33 years indicates that c.[1426 C > A; 1437 + 1 G > A] probably leads to complete loss of function [36].

Regarding the mutation c.1637-2A > G the absence of any normally spliced product conforms to the classic infantile course of Pompe disease in patient 2 who is homozygous for this mutation.

The disease causing effect of some of the novel missense mutations and of the 9 base pairs deletion was not directly proven, since no functional studies were carried out. Yet, their absence on 120 alleles of unaffected persons as well as patients together with the conservation of the affected amino acids among the twenty-two eutheria (Additional file 2) makes it very likely that they are disease causing. The substitution c.1802 C > T (p.Ser601Leu) is the third mutation discovered in this codon. Previously, c.1802 C > G (p.Ser601Trp) and c.1802 C > A (pSer601X) were reported as pathogenic sequence variations [37,38]. We found the c.2297A > G mutation in a patient with a childhood course of the disease compound heterozygous with the base exchange c.1561 G > A that leads to a total loss of enzyme activity [39]. These findings indicate that c.2297A > G allows for a low residual activity.

Nevertheless the effect of novel mutations should be assessed by functional studies. This would provide the highest insight in the functional effects of the sequence variations as well as the best basis for investigating genotype-phenotype correlation.

### Genetic variability

The comparison of the frequencies of 34 polymorphisms with reference populations revealed an association of the mutation c.-32-13 T > G with the haplotype [c.324 T, c.596A, c.668 G, c.1203 G, c.1327-18A, c.2040 + 20A, c.2338G]. Since in addition the changes c.271 G > A, c.1726 G > A, c.2065 G > A, c.2446 G > A and c.2780 C > T rarely appear in this cohort the common mutation seems to be associated with the haplotype DHRGEVVT (c.271 G, c.596A, c.668 G, c.1726 G, c.2065 G, c.2338 G, c.2446 G, c.2780 C) in the German population. This association is in line with previous findings in the European population and corroborates the hypothesis of a founder effect [18].

### Genotype-phenotype correlations

The primary effect of residual enzyme activity on the clinical course of Pompe disease can be confirmed [2,3,10,16,17,40]. All our classic infantile patients had virtually no enzyme activity due to two severe mutations. Residual activities in non-classic patients correlated with a later age of onset and slower disease progression. All subgroups harboured patients with very low activities. Yet, the highest values were found among the patients with an adult course of the disease. Thus, high residual activities protect against a severe course of disease. Current disease severity did not correlate with enzyme activity although proof of this correlation is difficult to show in such small cohorts with a broad range of current age.

As in previous studies, the most common c.-32-13 T > G mutation is associated with a milder course although there is a broad variability in the decline of locomotive and respiratory function [4,11,13,18]. This is underlined by the discovery of a c.-32-13 T > G homozygous patient. Since homozygous patients were only sporadically reported it can be assumed that the homozygous state of c.-32-13 T > G usually is none-penetrating [13,41,42].

Phenotypic variability points to disease modifying factors that obtain relevance when the underlying pathogenic mutations allow for appreciable residual activity [18] The broad range of enzyme activities in c.-32-13 T > G compound heterozygous patients that cannot be described to the nature of the mutations on the second allele speaks for factors modulating the expression of the GAA gene, alternative splicing or the maturation of the translational products [43,44]. Variation can also be envisaged to develop on the basis of qualitative and quantitative differences in the autophagy pathway that influence the lysosomal glycogen deposition [45].

The fact that three of four patients who had a temporal regression of symptoms where quite active might indicate that muscle training can ameliorate the course of disease [46]. Muscle regeneration decreases with age, but is enhanced by training and proper nutrition, especially in young people [47,48]. It is certainly worthwhile to further elucidate the regulation of muscle plasticity and regeneration in future studies.

## Conclusions

This study is one of the largest single-centre studies regarding phenotypes as well as genotypes in Pompe disease. The disease course is dominated by progressive locomotive and respiratory impairment in all patients and and hypertrophic cardiomyopathy in patients with classic infantile disease. The clinical spectrum strongly correlates with residual acid alpha-glucosidase activity. Residual activity of this enzyme is primarily determined by the severity of the pathogenic mutations on both GAA alleles and likely controlled by yet unknown modifying factors.

## Competing interests

Research was supported by Genzyme Corporation. R Hartung is a member of the European Pompe Registry Board and received compensation from Genzyme Corporation for participation in meetings, AJJ Reuser received consulting honorarium from Genzyme Corporation, M. Beck and E. Mengel received consulting honorarium, payment for lectures and meeting expenses from Genzyme Corporation, S. Gökce received payment for lectures from Genzyme Corporation.

## Authors’ contributions

AH performed mutation analysis, statistical analysis and wrote the manuscript, RH collected clinical data and designed the study. Dr AJJR reviewed the manuscript. Dr PH performed mutation analysis. Dr HR collected clinical data and reviewed the manuscript. Dr NK collected clinical data, SG collected clinical data. Prof. Dr. JP designed the study and reviewed the manuscript. Prof Dr CK performed cardiac evaluation. Dr. CL collected clinical data. Prof Dr MB designed the study and performed biochemical assays. Dr EM designed the study, performed biochemical assays, collected clinical data and reviewed the manuscript. All authors read, edited and approved the final version of the manuscript.

## Supplementary Material

Additional file 1 Protocol of mutation analysis. Click here for file

Additional file 2 Alignment of protein sequences surrounding missense mutations with orthologous enzymes of different eutheria. Click here for file

Additional file 3 Allele frequencies of polymorphisms in comparison with reference populations. Click here for file
