# Supplementary material for: A cross-sectional single-centre study on the spectrum of Pompe disease, German patients: molecular analysis of the GAA gene, manifestation and genotype-phenotype correlations
Source: Orphanet J Rare Dis. 2012 Jun 7;7:35. doi: 10.1186/1750-1172-7-35 (PMC3479421; doi:10.1186/1750-1172-7-35)
Supplement: Additional file 1 — Protocol of mutation analysis. [file 1750-1172-7-35-S1.doc]

**Additional file 1: Protocol of mutation analysis**

Genomic DNA and RNA were extracted from whole blood using the QIAamp® DNA Blood mini Kit (Qiagen, Hilden, Germany) and the QIAamp® RNA Blood mini Kit (Qiagen, Hilden, Germany), respectively. cDNA was synthesized using the Reverse Transcription System (Promega, Mannheim, Germany) as recommended by the manufacturer.

Long range PCRs of the GAA gene were performed using the primersets as listed in Table supplement 1. Each reaction was performed in a volume of 25 µl containing 80 mM Tris-HCl, 20 mM (NH_4_)_2_SO_4_, 1.5 mM MgCl_2_, 1 M Betain or 10 % by volume DMSO, 125-250 μM of each dNTP, 0.2-0.4 μM forward and reverse primer, 1-2.5 U Taq DNA polymerase and 50-100 ng DNA. The reaction was started with denaturation for 5 min at 94°C, followed by 40 cycles with denaturation for 20 sec at 94°C, annealing for 30 sec at 58-62°C and extension for 2 min at 72°C. They were followed by a final extension for 7 min at 72°C.

| **Exon** | **Forward primer sequence (5´→3´)** | **Product length ^a^**  **in bp** |
| --- | --- | --- |
|  | **Reverse primer sequence (5´→3´)** |  |
| 1 | CTC TGA CCC CAG AGG AAC C | 709 |
|  | GGA GGA CTA GAG GTC TGG GG |  |
| 2-3 | GGT TGA TGT CTC AGA GCT GC | 1487 |
|  | CCC TGC CGT GTG AGA AAT G |  |
| 4-5 | GTG CTC TCA GGC TCG TGT G | 478 |
|  | CAT GCG GAC CTC CAG TCT C |  |
| 4-8 | GTG CTC TCA GGC TCG TGT G | 1445 |
|  | AGA AAA CAT CCT CGG CGA C |  |
| 9-11 | GCC TCA TCC TCT CAC TGT CTC | 1262 |
|  | GCT TCT CAG AGA TGA GGG TG |  |
| 12-15 | CTG AAG AGG CAG CGA CCT G | 1563 |
|  | GCT GCC TGG GAG TTA CGT G |  |
| 16-17 | AGC AGA ATT CAG CCT CTT CC | 1013 |
|  | TGA TAA CCT ACA CTG CGG GG |  |
| 17-19 | AGA TGG AGA GCG TGG TTC C | 1394 |
|  | GTC CCA GCA TCC TCT GTT C |  |
| 20 | GTT AAG GTG ACC CGC ACT G | 605 |
|  | GTG CTG GGA ACA GAT GGA G |  |

^a^ expected product length

Table supplement 1: Primer sets used for amplification of genomic DNA and expected product length.

PCR products were purified and sequenced directly on an ABI 3100 Avant-Genetic Analyzer (Applied Biosystems, Weiterstadt, Germany). Amplified Fragments of gDNA were sequenced using the same primers as in PCR as well as nested primers (Table supplement 2). Sequencing of cDNA fragments was done with M13 primers as well as with the primers of PCR as nested primers in long fragments.

| **Primer** | **Primer sequence (5´→3´)** |
| --- | --- |
| GAA Ex1-F | CTC TGA CCC CAG AGG AAC C |
| GAA Ex1-R | GGA GGA CTA GAG GTC TGG GG |
| GAA Ex2-F | GGT TGA TGT CTC AGA GCT GC |
| GAA Ex2-R | GCC ATT GTC TGC TCA CAC C |
| GAA Ex3-F | AGG ACC TGA CCT GTC CTT GG |
| GAA Ex3-R | CCC TGC CGT GTG AGA AAT G |
| GAA Ex4-F | GTG CTC TCA GGC TCG TGT G |
| GAA Ex5-R | CAT GCG GAC CTC CAG TCT C |
| GAA Ex6-F | GAG AGA GCC TCA ACT CTC CG |
| GAA Ex7-R | CAT GTA GTC CAG GTC GTT CC |
| GAA Ex8-F | AGG TGG TGG AGA ACA TGA CC |
| GAA Ex8-R | AGA AAA CAT CCT CGG CGA C |
| GAA Ex9-F | GCC TCA TCC TCT CAC TGT CTC |
| GAA Ex9-R | ATG GCT CCT CAA ATC CCA C |
| GAA Ex10-F | ACT AAG AGT GAG GCT GCC C |
| GAA Ex11-R | GCT TCT CAG AGA TGA GGG TG |
| GAA Ex12-F | CTG AAG AGG CAG CGA CCT G |
| GAA Ex12-R | GCC CCA ACC TTG TAG GAC AG |
| GAA Ex13-F | GAC AGG GTT CCC GAG TGA C |
| GAA Ex14-R | ATT CCC AGG GGA GAG TCT TG |
| GAA Ex15-F | GAG AAG TGC AGC TCT CCC G |
| GAA Ex15-R | GCT GCC TGG GAG TTA CGT G |
| GAA Ex16-F | AGC AGA ATT CAG CCT CTT CC |
| GAA Ex16-R | GCC GGG ACT CAA CAC ATA C |
| GAA Ex17-F | AGA TGG AGA GCG TGG TTC C |
| GAA Ex17-R | TGA TAA CCT ACA CTG CGG GG |
| GAA Ex18-F | GCT GTA CCA GCC TAG CAT TC |
| GAA Ex18-R | CTA GTG GCA GGT AGC CAT CG |
| GAA Ex19-F | ATG CCA TCA TGA GTC CCT G |
| GAA Ex19-R | GTC CCA GCA TCC TCT GTT C |
| GAA Ex-20F | GTT AAG GTG ACC CGC ACT G |
| GAA Ex-20R | GTG CTG GGA ACA GAT GGA G |

Table supplement 2: Primers used for amplification and sequencing of genomic DNA fragments.
