# Supplementary material for: A cross-sectional single-centre study on the spectrum of Pompe disease, German patients: molecular analysis of the GAA gene, manifestation and genotype-phenotype correlations
Source: Orphanet J Rare Dis. 2012 Jun 7;7:35. doi: 10.1186/1750-1172-7-35 (PMC3479421; doi:10.1186/1750-1172-7-35)
Supplement: Additional file 2 — Alignment of protein sequences surrounding missense mutations with orthologous enzymes of different eutheria. [file 1750-1172-7-35-S2.doc]

**Additional file 2: Alignment of protein sequences surrounding missense mutations with orthologous enzymes of different eutheria**

| **Order** | **Family** | **Species** | **Sequence**  **c.701C>A** | **Sequence**  **c.1291_1299del**  **CTGCACCAG** | **Sequence**  **c.1703A>T** | **Sequence**  **c.1802C>T** | **Sequence**  **c.2297A>G** | **Sequence**  **c.2738C>G** |
| --- | --- | --- | --- | --- | --- | --- | --- | --- |
| Primates | Hominidae | Homo sapiens, mutant | GRVLLN**K**TVAPLF | PAMVQE**---**GGRRY | HQFLST**L**YNLHN | PFVISR**L**TFAGH | KAEVTG**C**FPLGT | LGVATA**R**QQVLS |
|  |  | Homo sapiens, wild type | GRVLLN**T**TVAPLF | PAMVQE**LHQ**GGRRY | HQFLST**H**YNLHN | PFVISR**S**TFAGH | KAEVTG**Y**FPLGT | LGVATA**P**QQVLS |
|  |  | Gorilla gorilla | GRVLLN**T**TVAPLF | PAMVQE**LHQ**GGRRY | HQFLST**H**YNLHN | PFVISR**S**TFAGH | KAEVTG**Y**FPLGT | LGVATA**P**QQVXS |
|  |  | Pan troglodytes | GRVLLN**T**TVAPLF | PAMVQE**LHH**GGRRY | HQFLST**H**YNLHN | PFVISR**S**TFAGH | KAEVTG**Y**FPLGT | LGVATA**P**QQVLS |
|  |  | Pongo pygmaeus | GRVLLN**T**TVAPLF | PAMVRE**LHQ**GGRRY | HQFLST**H**YNLHN | PFVISR**S**TFAGH | KAEVTG**Y**FPLGT | LGVATA**P**QQVLS |
|  | Cercopithecidae | Macaca mulatta | GRVLLN**T**TVAPLF | PAMVQE**LHQ**GGRRY | HQFLST**H**YNLHN | PFVISR**S**TFAGH | KAEVTG**Y**FPSGT | LGVATA**P**QQVLS |
|  | Tarsiidae | Tarsius syrichta | GRVLLN**T**TVAPLF | XXXXXXXXXXXXXX | HQFLST**H**YNLHN | XXXXXXXXXXXX | KAEVTG**Y**FPTGT | LGVGTA**P**QQVLS |
|  | Cheirogaleidae | Microcebus murinus | GRVLLN**T**TVAPLF | PAMVQE**LHQ**GGRRY | RQFLST**H**YNLHN | PFVISR**S**TFSGH | XXXXXXXXXXXX | LGVATA**P**QQVLS |
|  | Galagonidae | Otolemur garnettii | GRLLLN**T**TVAPLF | XXXXXXXXXXXXXX | HQFLST**H**YNLHN | PFIISR**S**TFAGH | KTEVTG**Y**FPSGT | LGVATA**P**QQVLS |
| Rodentia | Sciuridae | Spermophilus tridecemlineatus | GRVLLN**T**TVAPLF | PATVPQ**LHQ**SGRRY | HQFLST**H**YDLHN | PFVISR**S**TFAGH | XXXXXXXXXXXX | LGVDPA**P**QQVLS |
|  | Caviidae | Cavia porcellus | GRVLLN**T**TVAPLI | PATVHE**LHQ**GGRRY | HQFLST**H**YNLHN | PFVISR**S**TFAGH | KTQVTG**Y**FPLGT | LGVAQA**P**RQVLS |
|  | Muridae | Mus musculus | GRVLLN**T**TVAPLF | PDMVRE**LHQ**DGRRY | HQFLST**H**YNLHN | PFVISR**S**TFSGH | KTEVTG**Y**FPKGT | LGVATA**P**TQVLS |
|  | Heteromyidae | Dipodomys ordii | GWVLLN**T**TVAPLI | PDTVRE**LHQ**GGRRY | LQFLST**H**YNLHN | SFVISR**S**TFSGH | KDEVTG**Y**FPSGT | LGVSAA**P**QQVLS |
| Lagomorpha | Ochotonidae | Ochotona princeps | GRVLLN**T**SVAPLF | PTMVRE**LHE**GGRRY | RQFLST**H**YNLHN | PFVISR**S**TFAGH | KVNVTXXXXXXX | LGVATA**P**HQVLS |
| Artiodactyla | Bovidae | Bos taurus | GRVLLN**T**TVAPLF | PAMVQE**LHQ**GGRRY | HQFLST**H**YDLHN | PFVISR**S**TFAGH | KVEVTG**Y**FPQGT | LGVATA**P**QQVLC |
| Perissodactyla | Equidae | Equus caballus | GRVLLN**T**TVAPLF | PAMVQE**LHQ**GGRRY | QQFLST**H**YDLHN | PFVISR**S**TFAGH | RVEVTG**Y**FPLGT | LGVAVA**P**QQVLS |
| Carnivora | Felidae | Felis catus | GRVLXXXXXXXXX | XXXXXXXXXXXXXX | XXXXXXXXXXXX | XXXXXXXXXXXX | KVEVTG**Y**FPAGT | LGVAAA**P**KQVLS |
|  | Canidae | Canis familiaris | GRVLLN**T**TVAPLF | PAMVQE**LHR**GGRRY | RQLLST**H**YNLHN | PFVISR**S**TFAGH | KVEVTG**Y**FPAGT | LGVAAG**P**RQVLC |
| Chiroptera | Pteropodidae | Pteropus vampyrus | GRVLLN**T**TVAPLF | PAMVQE**LHQ**SGRRY | XXXXXXXXXXXX | XXXXXXXXXXXX | XXXXXXXXXXXX | LGVATA**P**QQVVS |
|  | Vespertilionidae | Myotis lucifugus | GRVLLD**T**AVAPLF | XXXXXXXXXXXXXX | RQFL-T**H**YNLHN | PFVISR**S**TFAGH | KVEVTG**Y**FPAGT | LGVASA**P**KQVLA |
| Cetacea | Delphinidae | Tursiops truncatus | GRVLLN**T**TVAPLF | PAMVQE**LHQ**SGRRY | HQFLST**H**YDLHN | PFVISR**S**TFAGH | KVEVTG**Y**FPRST | LGVATA**P**QQVVC |
| Eulipotyphla | Erinaceidae | Erinaceus europaeus | GRVLLN**T**TVAPLF | XXXXXXXXXXXXXX | RQFLST**H**YNLHN | PFIISR**S**SFSGH | RTEVTG**Y**FPAGT | LGVAIA**P**QQVLC |
| Proboscidea | Elephantidae | Loxodonta africana | XXXXLN**T**TVAPLF | XXXXXXXXXXXXXX | XXXXXXXXXXXX | PFVISR**S**TFASH | KAEVTG**Y**FPLGT | LGVATA**P**RQVLS |

xxx unknown

Conservation: 90-100%, 80-89%, 70-79%, 60-69%, <69%

Table supplement 3: Alignment of protein sequences surrounding missense mutations with orthologous enzymes of different eutheria
