# Supplementary material for: A cross-sectional single-centre study on the spectrum of Pompe disease, German patients: molecular analysis of the GAA gene, manifestation and genotype-phenotype correlations
Source: Orphanet J Rare Dis. 2012 Jun 7;7:35. doi: 10.1186/1750-1172-7-35 (PMC3479421; doi:10.1186/1750-1172-7-35)
Supplement: Additional file 3 — Allele frequencies of polymorphisms in comparison with reference populations. [file 1750-1172-7-35-S3.doc]

**Additional file 3: Allele frequencies of polymorphisms in comparison with reference populations**

| **Nucleotide exchange** | **dbSNP rs#** | **Protein effect** | **Allele frequency in %** | | |
| --- | --- | --- | --- | --- | --- |
|  |  |  | **Patients with**  **genotype**  **c.-32-13T/G**  **(48-54 alleles)** | **Patients with**  **genotype**  **c.-32-13T/T**  **(11-12 alleles)** | **Reference collective**  **(number of alleles, population)** |
| c.-367-157C>G |  | 5´-UTR | 17 | 0 | - |
| c.-367-127C>T | 2304850 | 5´-UTR | 25 | 33 | - |
| c.-260G>C | 2304849 | 5´-UTR | 21 | 33 | - |
| c.271G>A | 1800299 | p.Asp91Asn | 7 | 8 | 3,03 (2312, Swallow 1975) |
| c.324T>C | 1800300 | p.Cys108Cys | 39 | 67 | 77,6 (116, Hapmap-CEU) |
| c.510C>T |  | p.Asp170Asp | 6 | 0 | - |
| c.547-4C>G | 3816256 | - | 41 | 67 | 23,3 (120, Hapmap-CEU) |
| c.596A>G | 1042393 | p.His199Arg | 37 | 67 | 73,5 (102, Hapmap-CEU) |
| c.642C>T | 1800301 | p.Ser214Ser | 2 | 0 | 28,0 (184, CEPH) |
| c.668G>A | 1042395 | p.Arg223His | 39 | 67 | 76,7 (120, Hapmap-CEU) |
| c.852G>A |  | p.Ala284Ala | 2 | 0 | - |
| c.858+8_858+9  insAGCGGGC | 35373675 | - | 37 | 67 | - |
| c.858+30T>C | 2304845 | - | 37 | 67 | - |
| c.921A>T | 1800303 | p.Ala307Ala | 0 | 0 | 6,0 (184, CEPH) |
| c.955+12G>A | 2252455 | - | 37 | 67 | - |
| c.1203G>A | 1800304 | p.Gln401Gln | 43 | 67 | 62,0 (92, AoD Caucasian) |
| c.1265G>A | 2229224 | p.Arg422Gln | 7 | 0 | - |
| c.1327-18A>G | 2278619 | - | 43 | 67 | 78,3 (120, Hapmap-CEU) |
| c.1374C>T | 1800305 | p.Tyr458Tyr | 2 | 0 | 7,0 (184, CEPH) |
| c.1438-19G>C | 2304844 | - | 39 | 67 | 35,0 (92, AoD Caucasian) |
| c.1551+49C>A | 2304843 | - | 39 | 67 | - |
| c.1581G>A | 1042396 | p.Arg527Arg | 24 | 42 | 31,0 (116, Hapmap-CEU) |
| c.1726G>A | 1800307 | p.Gly576Ser | 6 | 0 | 0,0 (120, Hapmap-CEU) |
| c.1888+21G>A | 2304837 | - | 4 | 0 | 0,9 (114, Hapmap-CEU) |
| c.2040+20A>G | 2304836 | - | 39 | 67 | 73 (222, Hapmap-CEU) |
| c.2065G>A | 1800309 | p.Glu689Lys | 4 | 0 | 2,5 (120, Hapmap-CEU) |
| c.2133A>G | 1800310 | p.Thr711Thr | 11 | 17 | 28,3 (182, CEPH) |
| c.2331+20G>A | 2304832 | - | 42 | 71 | - |
| c.2331+24T>C | 2304831 | - | 6 | 0 | - |
| c.2338G>A | 1126690 | p.Val780Ile | 33 | 58 | 79,2 (120, Hapmap-CEU) |
| c.2446G>A | 1800314 | p.Val816Ile | 0 | 0 | 0,0 (184, CEPH) |
| c.2553G>A | 1042397 | p.Gly851Gly | 31 | 64 | - |
| c.2780C>T | 1800315 | p.Thr927Ile | 0 | 0 | 0,8 (118, Hapmap-CEU) |
| c.*+91G>A | 2229221 | 3´-UTR | 2 | 0 | 8,8 (114, Hapmap-CEU) |

| Hapmap-CEU: | DNA probes from Utah residents with Northern and Western European ancestry from the collection of the Centre de’Etude du Polymorphism Humain (CEPH).[1, 2] |
| --- | --- |
| CEPH: | DNA probes of individuals descendend from Utah (93%), France (4%) und Venezuela (3%) from the collection of the Centre de’Etude du Polymorphism Humain (CEPH).[1] |
| AoD Caucasian: | DNA-probes from Caucasian persons.[1] |
| Swallow 1975: | Swallow DM, Corney G, Harris H et al. Acid alpha-glucosidase: a new polymorphism in man demonstrable by 'affinity' electrophoresis. *Ann. Hum. Genet*. 1975;38:391–406. |

Table supplement 4: Allele frequencies of polymorphisms in comparison with reference populations

[1] NCBI: **SNP´s** [http://www.ncbi.nlm.nih.gov/SNP/snp_ref.cgi?showRare=on&chooseRs=all&locusId=2548&mrna=NM_000152.3&ctg=NT_024871.11&prot=NP_000143.2&orien=forward&refresh=refresh, accessed 22 Mar 2009].

[2] Broad Institute: **HapMap Data Rel 27 PhaseII+III, Feb09, on NCBI B36 assembly, dbSNP b126: chr17:75689950.75708273** [http://www.hapmap.org/cgi-perl/gbrowse/hapmap27_B36/, accessed 7 May 2009].
